# Human α10 nicotinic acetylcholine receptor subunits assemble to form functional receptors

**DOI:** 10.1016/j.jbc.2025.108182

**Published:** 2025-01-10

**Authors:** Bassel Tekarli, Layla Azam, Arik J. Hone, J. Michael McIntosh

**Affiliations:** 1 School of Biological Sciences, University of Utah, Salt Lake City, Utah, USA; 2 MIRECC, George E. Whalen Veterans Affairs Medical Center, Salt Lake City, Utah, USA; 3 Department of Psychiatry, University of Utah, Salt Lake City, Utah, USA; 4 George E. Whalen Veterans Affairs Medical Center, Salt Lake City, Utah, USA

**Keywords:** alpha10, nicotinic, acetylcholine, conotoxin, RIC-3, RgIA-5474, RgIA-4, [V11L;V16D]ArIB

## Abstract

Nicotinic acetylcholine receptors (nAChRs) are pentameric ligand-gated ion channels. In mammals, there are 16 individual nAChR subunits allowing for numerous possible heteromeric compositions. nAChRs assembled from **α**7 or **α**9 subunits will form homopentamers. In contrast, the structurally related **α**10 nAChR subunit has historically been thought to require **α**9 subunits for function. Recently, however, strychnine was shown to enable the expression of human **α**10 nAChRs in *Xenopus laevis* oocytes or mammalian cells, prompting a re-examination of whether the human **α**10 subunit can self-assemble in the absence of strychnine. In the present study, acetylcholine-evoked ionic currents were obtained by co-expression of human **α**10 nAChR subunits with the transmembrane protein resistance to inhibitors of cholinesterase-3 (RIC-3) in *Xenopus* oocytes. Furthermore, the creation of a gain-of-function reporter mutation, V13′T, in the second transmembrane domain demonstrated that **α**10 subunits can self-assemble in the presence or absence of RIC-3. The antagonist sensitivity of the homomeric **α**10 nAChR is distinct from that of the closely related **α**7 and **α**9**α**10 subtypes. **α**10 homomers were blocked by **α**-bungarotoxin but were insensitive to **α**-conotoxin [V11L;V16D]ArIB and RgIA-5474, which potently block **α**7 nAChRs and **α**9**α**10 nAChRs, respectively. These studies yield insight into the assembly of functional human **α**10 homomers and provide tools for the development of **α**10 -nAChR-selective ligands.

Ligand-gated ion channels are a large group of neurotransmitter receptors central to signal transduction. These receptors are composed of multiple subunits that assemble to form homo- or heteromeric complexes. The Cys-loop receptor superfamily of ligand-gated ion channels includes nicotinic acetylcholine, gamma-aminobutyric acid type A, serotonin (5-HT_3_), zinc-activated ion channel, and glycine receptors. Nicotinic acetylcholine receptors (nAChRs) in the central and peripheral nervous systems mediate reward, attention, and pain functions, and nAChRs in immune cells modulate inflammatory cytokine release (1, 2). Mammalian nAChRs are pentamers assembled from α1-α7, α9, α10 subunits, β1-β4 and γ, δ, and ε subunits (3). The ability to form numerous unique subunit permutations contributes to diverse pharmacological and physiological functions. In theory, any permutation of five subunits can form a pentamer. However, decades of investigation have indicated that the association of specific subunits may be far more restricted, and the rules of subunit assembly are not well understood. For example, α1, β1, and δ subunits will combine with either an ε subunit having a stoichiometry (α1)_2_β1δε (the adult muscle nAChR) or a γ subunit (α1)_2_β1δγ (a nAChR subtype found in fetal and denervated muscle). However, these same nAChR subunits have not been found to combine with any other α or β subunits. Separately, heteropentamers can form from α2-α7 and β2-β4 subunits with different stoichiometries adding an additional layer of complexity. For example, (α4)_2_(β2)_3_ and (α4)_3_(β2)_2_ form receptors with high and low affinity for acetylcholine (ACh), respectively (4).

α7 and α9 nAChRs can assemble as homopentamers (5). The α10 subunit was the last discovered member of the mammalian nAChR subunit gene family. Chick α10 will form a homopentamer (6). By contrast, the mammalian α10 subunit, since its discovery in 2001, has been believed to require an α9 subunit for expression. The consensus in the field has held that mammalian α10 subunits cannot form a homomer.

Recently, however, our group determined that exposure to certain alkaloids, including strychnine, brucine, and methyllycaconitine can enable the function of homomeric α10 mammalian nAChRs (7). Similar results with strychnine were found when α10 subunits were expressed in a mammalian cell line (8). These findings raise the possibility that α10 subunits might assemble naturally without the α9 subunit. Examination of the expression requirements of non-α10 nAChRs provides clues as to how this may occur. For instance, there is precedent for protein chaperones, including resistance to inhibitors of cholinesterase-3 (RIC-3), and nicotinic acetylcholine receptor regulator transmembrane protein 35a (NACHO), to facilitate the expression of homomeric α7 and other nAChR subtypes (9, 10). Similarly, an endogenous chaperone not present in cellular expression systems may be required for homomeric α10 nAChR expression.

nAChRs containing α10 subunits modulate the release of cytokines and form part of the cholinergic-anti-inflammatory system (2, 11). Pharmacological agents that act on these α10-containing nAChRs are analgesic in animal models of neuropathic pain (12, 13). However, the subunit composition of these receptors has not been fully elucidated. The present study aimed to determine whether α10 subunits will assemble as homomeric receptors in the absence of an exogenous ligand.

## Results

### The nAChR chaperone protein RIC-3 enables the formation of **α**10 nAChR homomers

**Figure 1:**
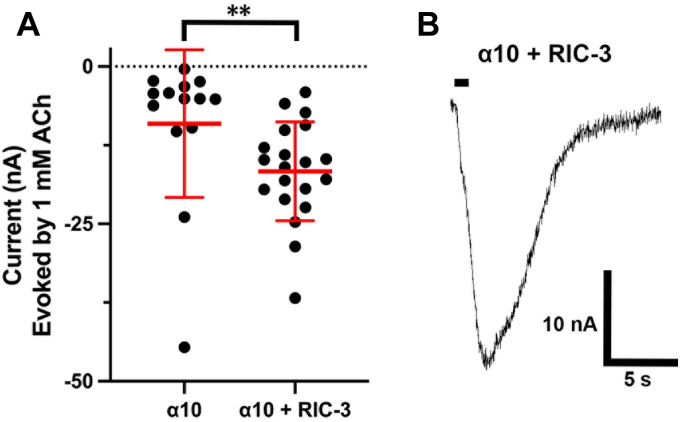
**RIC-3 enables α10 nAChR expression.***Xenopus laevis* oocytes were injected with cRNA encoding the α10 nAChR subunit then incubated at 16 °C for 3 days. Oocytes from both conditions were voltage-clamped at −70 mV and pulsed with ACh (1 mM) for 1 s. *A*, oocytes injected with both α10 nAChR and RIC-3 cRNA had significantly larger responses to ACh compared to oocytes injected with only α10 with mean responses of −17 ± 8 (n = 20) and −9 ± 12 (n = 14) respectively; ∗∗*p* < 0.01. Statistical significance was assessed by the Mann–Whitney test. *B*, the current trace of α10 and RIC-3 containing oocyte in response to 1 mM ACh. The error bars and “±” indicate SD. The horizontal bar indicates the application of a 1 s pulse of ACh into the oocyte chamber.

Protein chaperones including RIC-3 and NACHO have been reported to facilitate or be required for functional expression of some nAChR subtypes including homomeric α7 nAChRs (9, 10). RIC-3 is expressed in immune cells and neurons whereas NACHO is a neuronal-ER resident protein (10). Functional α10-containing nAChRs have thus far only been identified in non-neuronal cells, including immune cells. We, therefore, examined RIC-3 as a potential chaperone to enable α10 nAChR subunits to assemble as functional nAChR homomers in the absence of alkaloid ligands. Co-expression of human α10 and RIC-3 in *Xenopus laevis* oocytes enabled functional expression of α10 nAChRs (Fig. 1).

### ACh-evoked current in α10 and RIC-3-injected oocytes are inhibited by **α**-bungarotoxin but not by RgIA-5474

**Figure 2:**
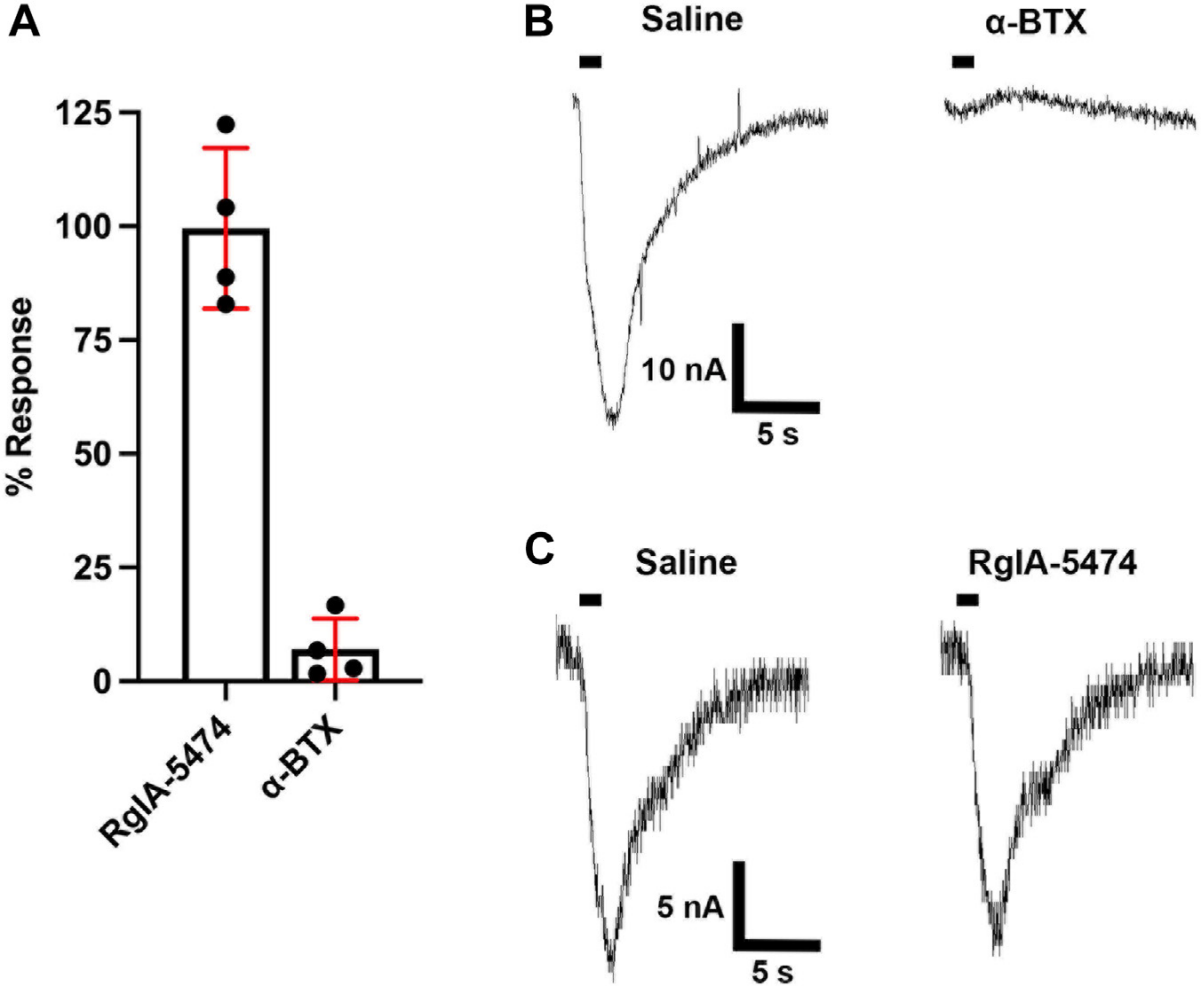
**α10 nAChRs are inhibited by α-bungarotoxin but not by RgIA-5474.***Xenopus laevis* oocytes were injected with cRNA encoding for α10 nAChRs and RIC-3, then incubated at 16 °C for 3 days. ACh-evoked current amplitudes were voltage-clamped at −70 mV and pulsed with ACh (1 mM) for 1 s. Currents were assessed in saline, and then compared to currents after exposure to α-bungarotoxin or RgIA-5474. *A*, Scatter plot of percent response to ACh after application of antagonist. *B*, ACh-evoked currents mediated by α10 nAChRs before and after a 5-min static bath of α-bungarotoxin (10 μM), representative of 5 trials. *C*, traces of α10 nAChRs before and after 5-min of acute perfusion of RgIA-5474 (100 nM), representative of 4 trials. The horizontal bar indicates the application of a 1 s pulse of ACh into the oocyte chamber.

Selective toxin-based peptides were used to assess the antagonist pharmacology of human α10 nAChRs. The snake venom-derived α-bungarotoxin completely blocked the nAChRs. In contrast, RgIA-5474, a selective conopeptide that blocks α9 homomers and α9α10 heteromers (14) failed to reduce the ACh-induced current when applied to α10-expressing oocytes (Fig. 2). Thus, the closely related α9, α9α10, and α10 nAChRs can be distinguished based on their sensitivity to these peptides. Each of the α9, α9α10, and α10 nAChRs subtypes are sensitive to α-bungarotoxin while only α10 is resistant to RgIA-5474.

### [V13′T]**α**10 subunits assemble as functional homomers

**Figure 3:**
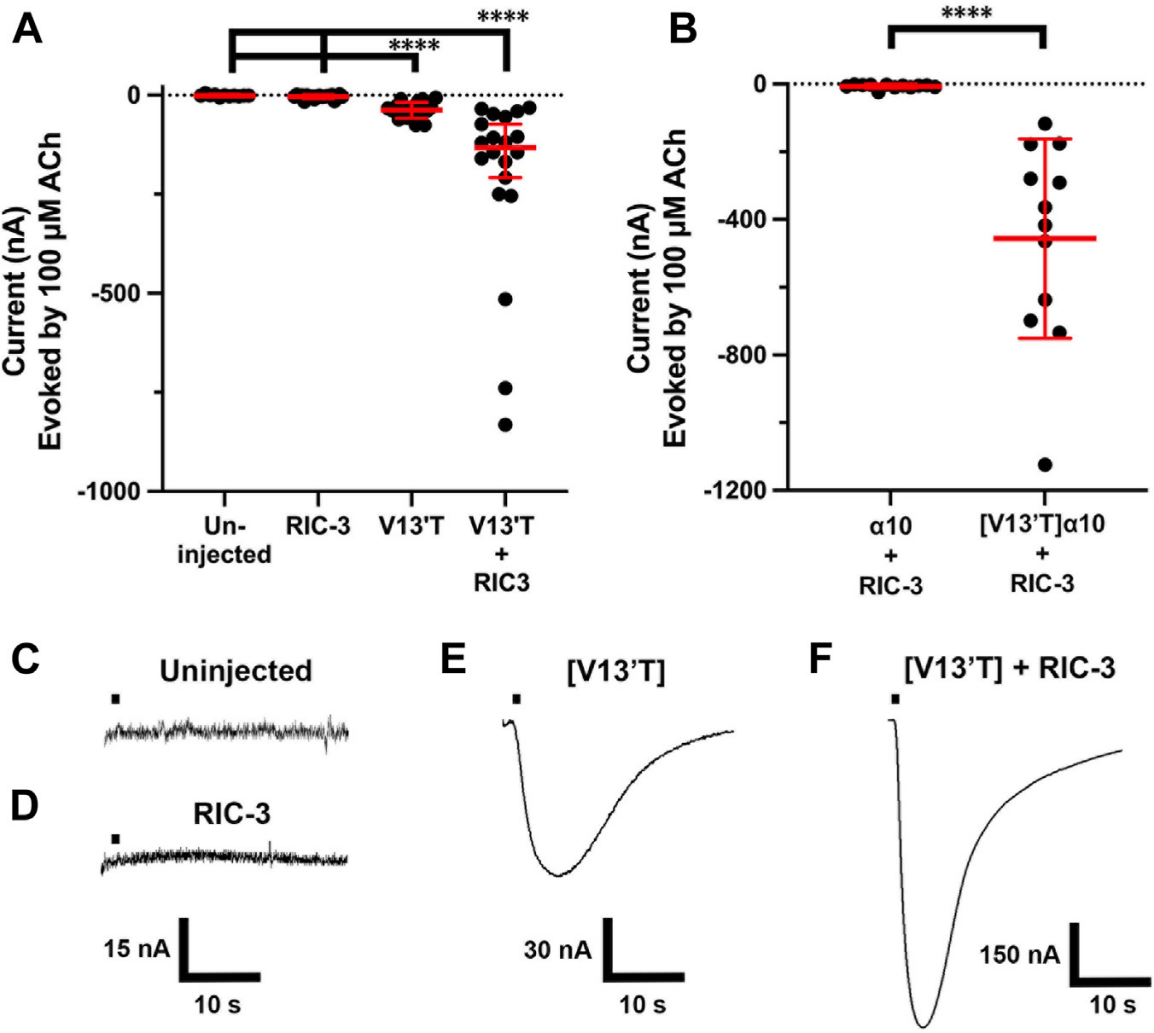
**Human [V13′T]α10 nAChR subunits form functional receptors**. *Xenopus* oocytes were injected in various combinations of cRNAs encoding for the RIC-3 chaperone, α10 nAChRs, or [V13′T]α10 nAChRs. As described in *Experimental Procedures*, responses to 1-s pulses of 100 μM ACh were assessed by voltage clamp electrophysiology at −70 mV. *A*, oocytes were injected with cRNA encoding for [V13′T]α10 nAChR subunits, RIC-3, or both. [V13′T]α10 nAChRs demonstrated pronounced currents with a mean response of −38 ± 20 nA. These nAChR current amplitudes were enhanced to −208 ± 227 nA when co-injected with RIC-3 cRNA. Uninjected oocytes (n = 10) and oocytes injected with RIC-3 alone (n = 20) did not produce currents. *B*, oocytes were injected with cRNA for either α10 or [V13′T]α10 subunits in combination with RIC-3. Oocytes injected with α10 nAChR subunits had a mean ACh-induced response of −7 ± 6 nA and a range of −2 to −24 nA (n = 12); oocytes injected with human [V13′T]α10 cRNA had a mean response of −456 ± 294 nA with a range of −117 to −1120 nA (n = 12); ∗∗∗∗*p* < 0.0001. Statistically significant measurements were assessed using the Mann–Whitney test. The error bars and “±” indicate SD. *C*, the current trace of uninjected oocytes. *D–F*, current traces of oocytes containing [V13′T]α10 nAChRs, RIC-3 chaperones, or both. The horizontal bar indicates the application of a 1 s pulse of ACh into the oocyte chamber.

As a separate approach to assessing whether α10 subunits can self-assemble as functional homomers, we introduced a gain-of-function mutation in the α10 subunit. Previous reports indicated that a V13′T substitution in the transmembrane-2 (TM2) domain of the human α10 subunit enhances the function of α9α10 heteromers (15). Therefore, we introduced this V13′T substitution in the α10 subunit and assessed ACh-evoked responses compared to those in oocytes expressing wild-type α10 subunits. The V13′T substitution significantly increased current amplitudes compared to wild-type controls (Fig. 3).

Given that co-expression with RIC-3 and the V13′T substitution each enhanced α10 nAChR expression/function, we assessed whether RIC-3 would potentiate the functional expression of [V13′T]α10. When oocytes were injected with cRNAs for [V13′T]α10 and RIC-3, the ACh-evoked responses were significantly larger than those injected with cRNA for [V13′T]α10 alone (Fig. 3). Given the robust enhancement of ACh-evoked responses, RIC-3 was used together with the [V13′T]α10 construct in all subsequent experiments.

### [V13′T]**α**10 current responses are not due to endogenous oocyte receptors

*X.laevis* oocytes are known to express endogenous receptors and ion channels on their cell membrane surface (16). Therefore, assessing these endogenous receptors' impact, or lack thereof, when measuring currents produced by exogenous nAChRs is important. nAChRs are non-selective cation channels. Among the endogenous receptors expressed on the cell surface, Ca^2+^-activated Cl⁻ channels can contribute to ACh-induced currents due to the influx of Ca^2+^ through the nAChR; these currents are eliminated when Ca^2+^ is removed from the perfusion solution (17). Oocytes expressing [V13′T]α10 nAChRs were examined in a saline solution containing either Ca^2+^ or Ba^2+^. Results indicate that the absence of Ca^2+^ did not substantially reduce ACh-evoked currents. This indicates that the contribution of Ca^2+^-activated chloride channels is small to absent.

**Figure 4:**
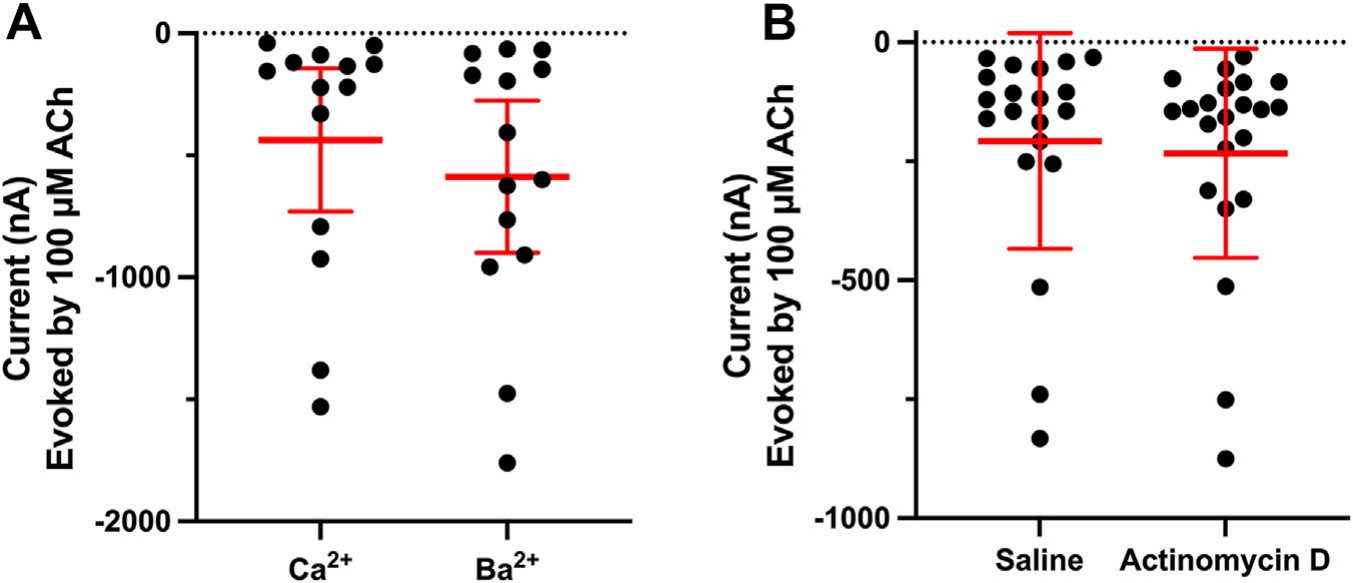
**ACh evoked currents in [V13′T]α10 nAChR expressing oocytes persist in the absence of Ca**^**2+**^**and when oocytes are incubated in actinomycin D.** cRNA encoding for [V13′T]α10 nAChRs was injected into *Xenopus laevis* oocytes. Oocytes were voltage-clamped at −70 mV and pulsed with ACh (100 μM) for 1 s. *A*, oocytes were perfused with saline containing either Ca^2+^ or Ba^2+^. [V13′T]α10-expressing oocytes had a mean ACh-induced response of −437 ± 508 nA in the presence of calcium and −587 ± 539 nA in the presence of barium. (n = 12), *p* > 0.05. *B*, immediately after injection of [V13′T]α10 nAChR cRNA, oocytes were incubated in an actinomycin D solution (1 mM) or saline for 2 days. The mean response was not significantly different between oocytes incubated with the presence of actinomycin D (−233 ± 219 nA) and without actinomycin D (−208 ± 227 nA); n = 20, *p* > 0.05. Values shown are means ± SD. Statistical significance was assessed by the Mann–Whitney test. The error bars and “±” indicate SD.

Separately, it is possible that *X. laevis* oocytes may express endogenous nAChR subunits that co-assemble with α10. We inhibited endogenous oocyte RNA transcription with actinomycin D, a DNA-dependent RNA polymerase inhibitor, to control for this possible variable. Oocytes expressing [V13′T]α10 nAChRs that were pre-treated with actinomycin D did not differ in the magnitude of their ACh-induced currents from untreated controls (Fig. 4). Thus, human α10 expression in oocytes is independent of endogenously expressed nAChR subunits.

### Nicotine and choline are partial agonists of [V13′T]**α**10

**Figure 5:**
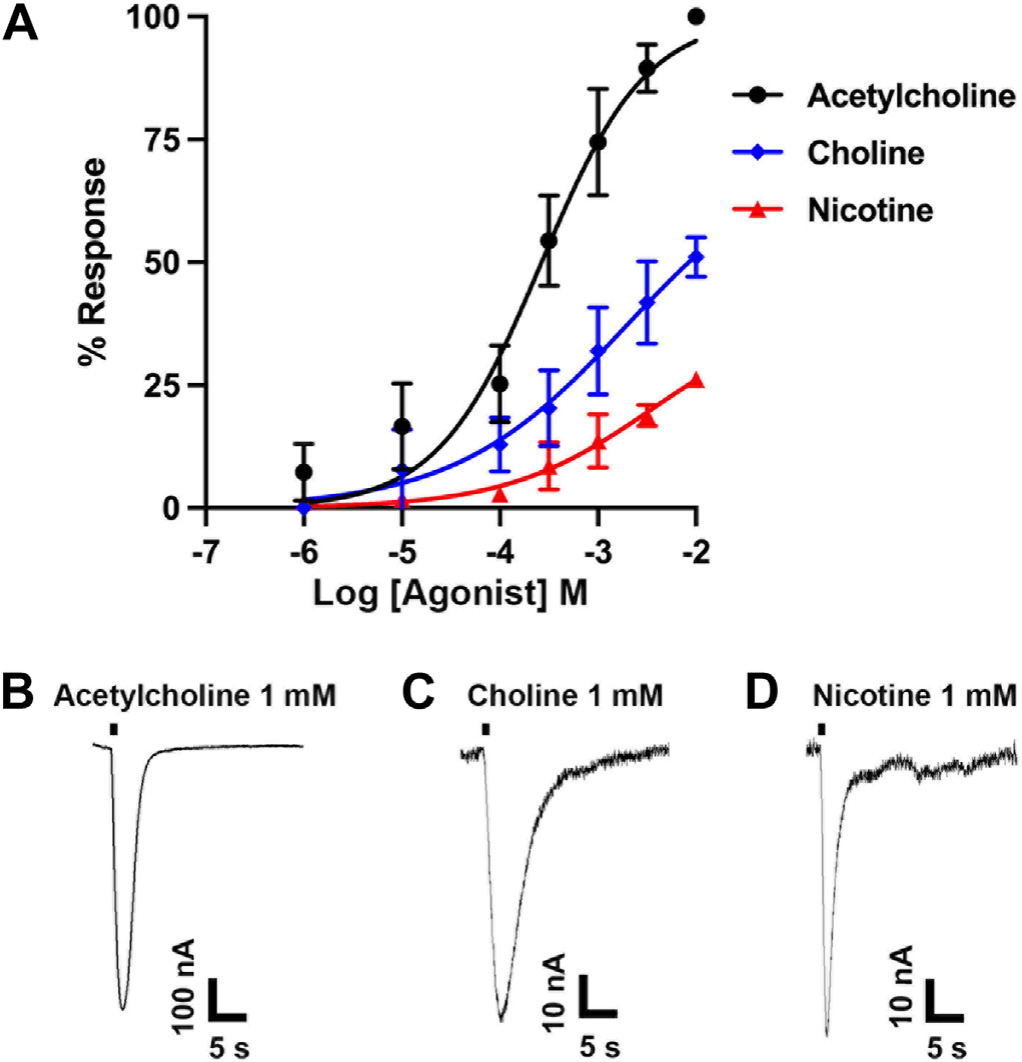
**Acetylcholine (ACh), choline, and nicotine induce currents in [V13′T]α10 nAChR expressing oocytes.** Oocytes were assessed by voltage-clamp electrophysiology for agonist-induced responses *A*, concentration-response curves of [V13′T]α10 nAChRs. ACh had an EC_50_ of 0.27 mM (0.21–0.33) with a Hill slope of 0.82 (0.67–1.01) (n = 7). Choline and nicotine were partial agonists with EC_50_s of 0.20 mM (0.15–0.28) and 0.35 mM (0.28–0.46), with Hill slopes of 0.49 (0.41–0.60), 0.59 (0.49–0.69) respectively (n = 6). *B–D*, current traces of [V13′T]α10 nAChRs activated by 1 mM ACh, choline, and nicotine. The error bars in (A) indicate SD and the values in parenthesis indicate 95% CI. Acetylcholine-induced currents were significantly larger than those induced by either choline or nicotine at all concentrations tested (see Table 1). The horizontal bar indicates the application of a 1 s pulse of ACh into the oocyte chamber.

Next, we determined concentration-response curves for ACh, nicotine, and choline. ACh had an EC_50_ of 0.27 mM. Both choline and nicotine had lower efficacy than ACh. Choline was more efficacious than nicotine evoking greater average currents at all tested concentrations (Fig. 5).

### Antagonist pharmacology of **α**9, **α**9**α**10, **α**7, and [V13′T]**α**10 nAChRs

**Figure 6:**
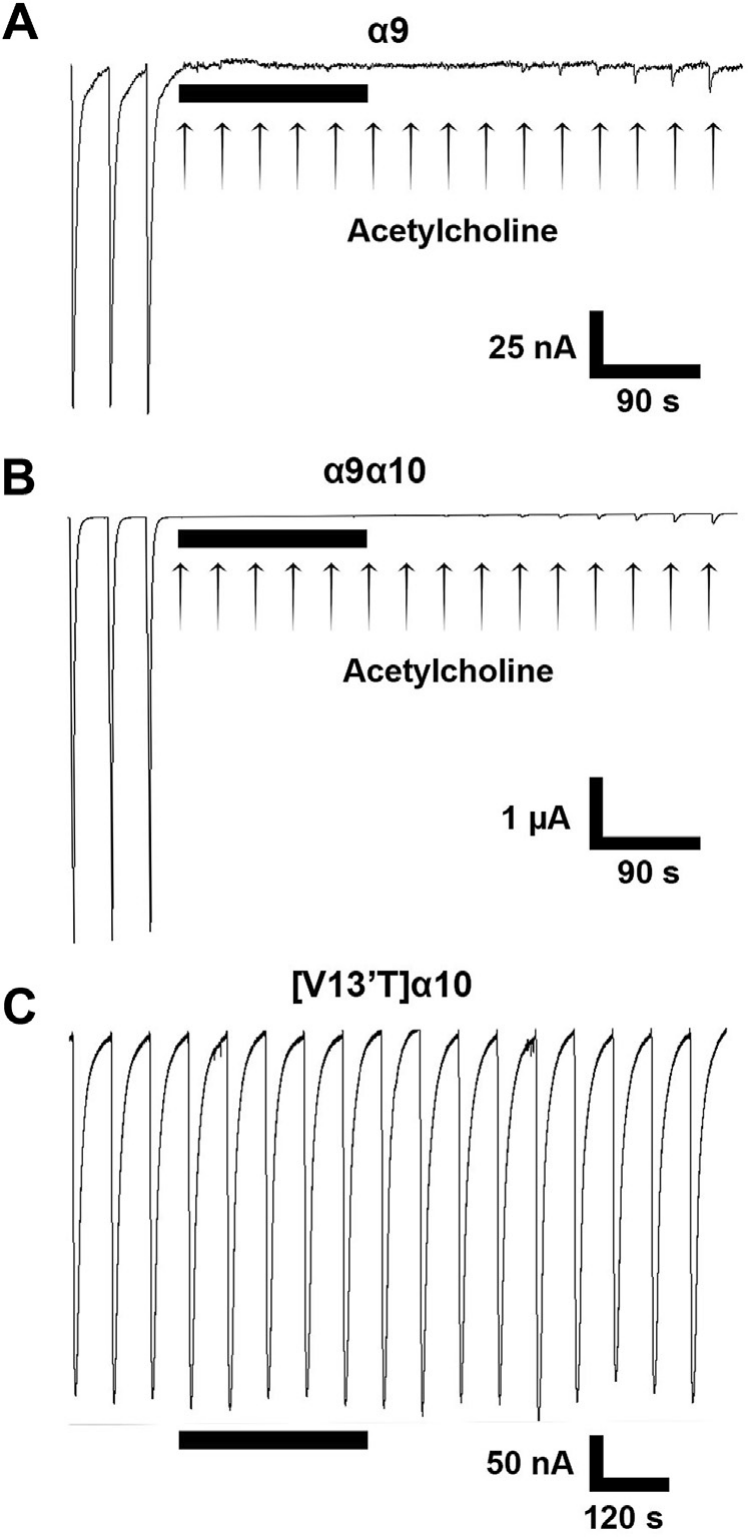
**[V13′T]α10 nAChRs are resistant to block by RgIA-5474.***Xenopus laevis* oocytes were injected with cRNA encoding nAChR subunits α9, α9, and α10, or [V13′T]α10. Oocytes were voltage-clamped at −70 mV and pulsed with 1-s of 100 μM ACh in the absence of and then in the presence of RgIA-5474. Currents evoked by ACh on α9 (*A*) and α9α10 (*B*) nAChRs were potently blocked by RgIA-5474, whereas RgIA-5474 failed to block [V13′T]α10 (*C*). Note that block by RgIA-5474 of α9 and α9α10 nAChRs is only slowly reversed upon washout of RgIA-5474 (n = 5). The horizontal black bar represents the period of RgIA-5474 perfusion and *arrows* indicate ACh pulses.

**Figure 7:**
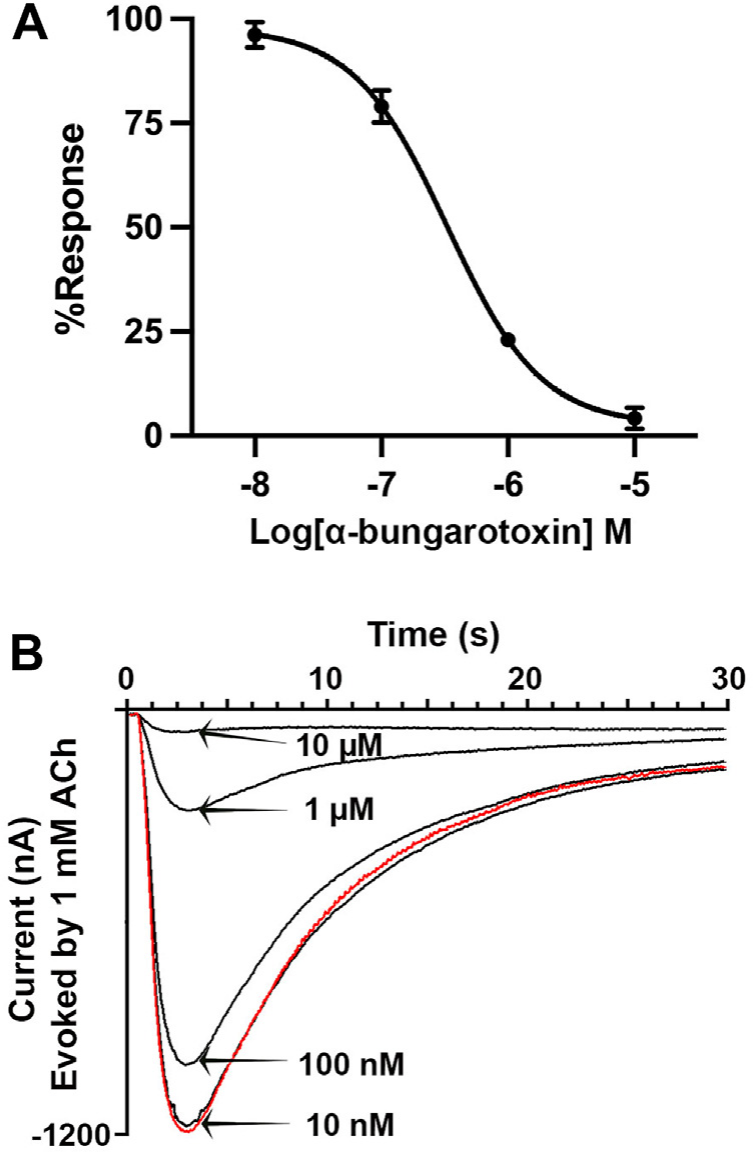
**α-Bungarotoxin potently inhibits [V13′T]α10 nAChRs.***Xenopus laevis* oocytes were injected with cRNA for [V13′T]α10 and voltage clamped at −70 mV; oocytes were pulsed with 1 mM ACh for 1 s. *A*, concentration-response curve for α-bungarotoxin induced block of ACh-evoked currents. The IC_50_ was 331 (273–400) nM with a Hill slope of −1.17 (−1.36 to −1.00) (n = 4). The error bars indicate SD and the values in parenthesis indicate 95% CI. *B*, superimposed ACh-induced currents when exposed to the indicated concentrations of α-bungarotoxin. The red trace indicates the response measured before α-bungarotoxin exposure.

**Figure 8:**
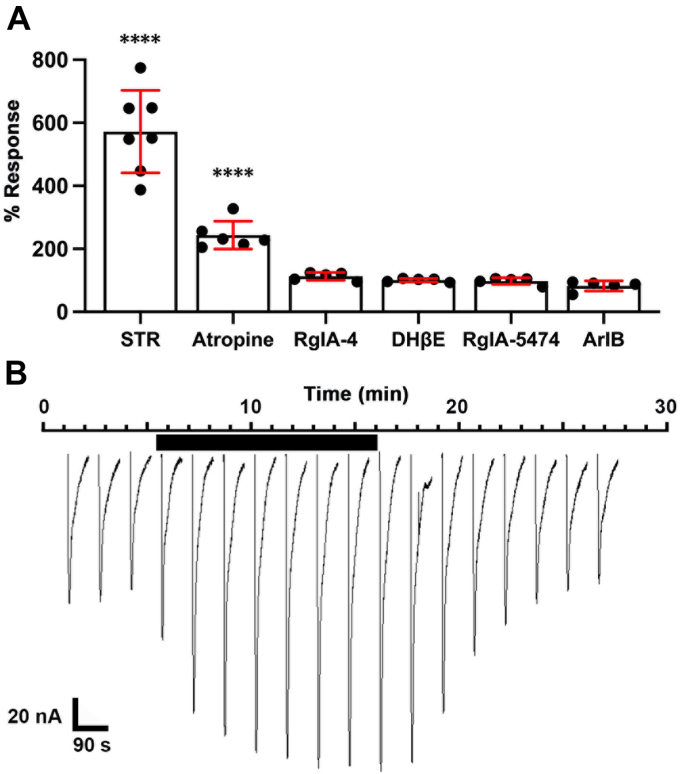
**ACh-induced currents are potentiated by atropine and strychnine.** [V13′T]α10 expressing oocytes were exposed to ACh in the presence and absence of strychnine (STR) (1 μM) or other indicated ligands (3 μM) for 10 min. *A*, oocytes were exposed to 1-s pulses of ACh (100 μM) at 90-s intervals. Responses to the indicated ligands are indicated as a percent of the ACh response obtained at baseline. ACh-evoked responses were potentiated to 230 ± 44% (n = 6) when exposed to atropine and 572 ± 131% (n = 7) when exposed to strychnine. The other compounds listed showed no significant activity. The concentration of RgIA-4, RgIA-5474, and dihydro-β-erythroidine (DHβE) was 3 μM and [V11L,V16D]ArIB was 1 μM (n = 5). Statistical significance was assessed by the Students *t*-test as described in the *Experimental Procedures*. *B*, atropine effects have a rapid onset and reversal. A baseline response to ACh pulses was established, followed by a 10-min perfusion of atropine (3 μM). Atropine was then washed out.

We examined the effects of known nAChR antagonists on [V13′T]α10 homomers, focusing on antagonists that target the closely related nAChR subtypes α9, α9α10, and α7. RgIA-5474 is a potent and selective inhibitor of α9 and α9α10 nAChRs (14). α9, α9α10, or [V13′T]α10 nAChRs were expressed in different oocytes and assessed for inhibition by RgIA-5474 (3 μM). Acetylcholine-induced currents from α9 and α9α10 nAChRs were inhibited by RgIA-5474 (Fig. 6). By contrast, ACh-evoked currents of [V13′T]α10 nAChRs showed no significant inhibition when exposed to the same concentration of RgIA-5474 (n = 6). Thus, RgIA-5474 can effectively discriminate not only between α9 and α10 homomers but also between α9α10 heteromers and α10 homomers. α-Bungarotoxin inhibited ACh-induced currents in [V13′T]α10 nAChRs with an IC_50_ of 331 nM (Fig. 7). α-Conotoxin [V11L,V16D]ArIB that completely blocks homomeric α7 nAChRs with low nM potency (18, 19) showed no significant inhibition of a9a10 nAChRs at 1 μM (Fig. 8).

### Alkaloids atropine and strychnine enhance ACh-induced activation of [V13′T]**α**10 nAChRs

Atropine is a classical muscarinic antagonist that is known to interact with nicotinic acetylcholine receptors containing α9 or α10 subunits (7, 20). Strychnine, the potent glycine receptor antagonist, also modulates nicotinic receptors and has been shown to enable functional expression of wild-type α10 homomers in oocytes (7). To examine the modulatory effects of atropine and strychnine, [V13′T]α10 homomers were expressed in oocytes and evaluated by a two-electrode voltage clamp (TEVC). ACh-induced activation of the channel was measured in the presence or absence of either alkaloid. Atropine and strychnine both demonstrated rapid, significant potentiation of ACh-evoked currents (Fig. 8). Strychnine and atropine enhanced responses by 572 ± 131% (n = 7) and 230 ± 44% (n = 6), respectively. ACh-evoked responses were not significantly altered by exposure to RgIA-4, RgIA-5474, or dihydro-β-erythroidine (Fig. 8).

### [V13′T]**α**10 nAChR activity is enhanced by strychnine at low concentrations and reduced at high concentrations

**Figure 9:**
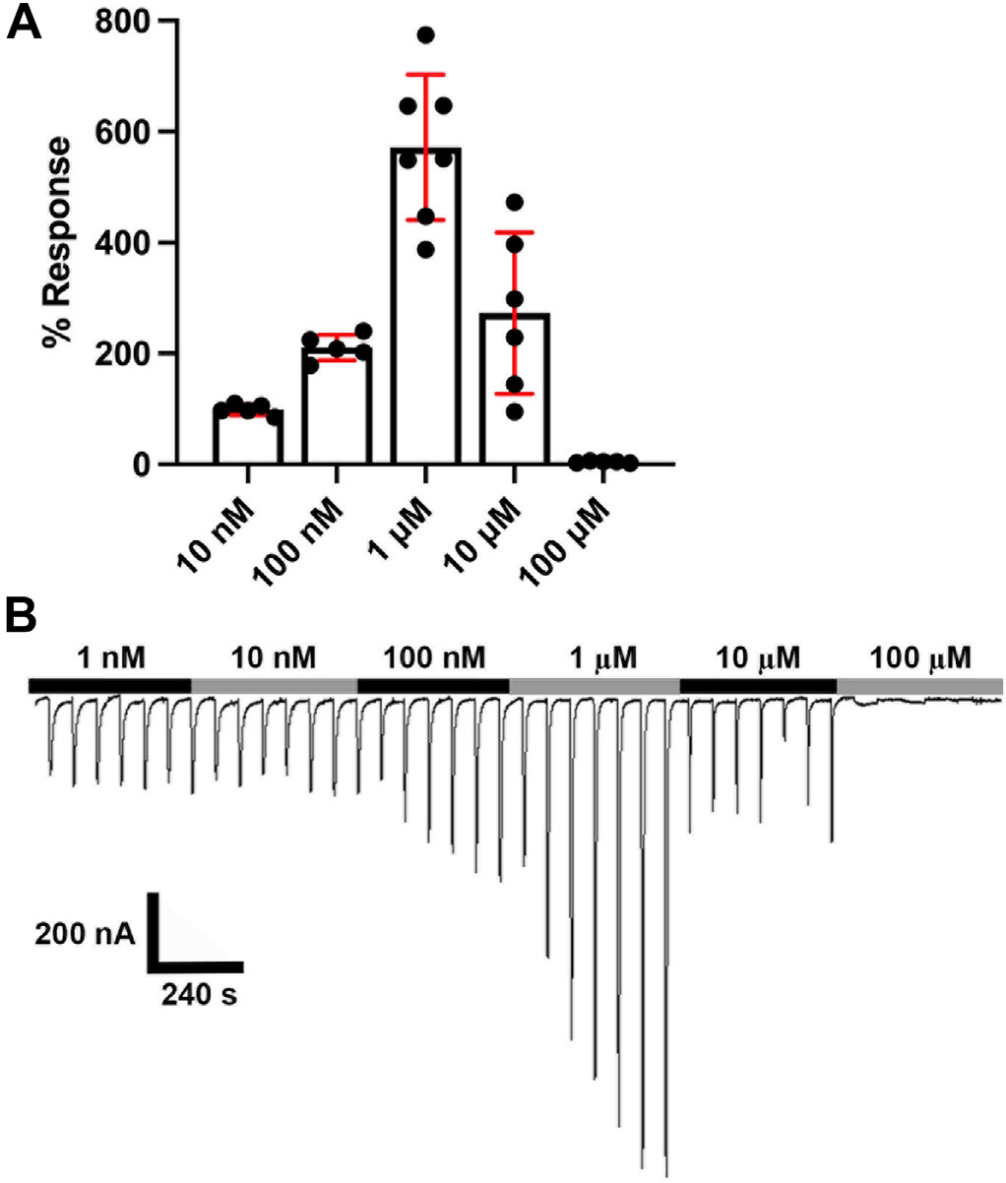
**Concentration-dependent effects of strychnine.***Xenopus laevis* expressing [V13′T]α10 nAChRs were voltage clamped at −70 mV, and the responses to ACh in the presence and absence of strychnine were measured. Oocytes were exposed to 1-s pulses of ACh (100 μM) for ∼ 20 min until a stable baseline response was achieved. Strychnine was then perfused for 10 min at the indicated concentrations. *A*, The responses to 10 nM to 100 μM strychnine were 98.8 ± 9.6% (10 nM), 210 ± 23.2% (100 nM), 572 ± 131% (1 μM), 273 ± 146% (10 μM), and 4.2 ± 1.8% (n = 5–7). The error bars and “±” indicate SD. *B*, representative current traces of [V13′T]α10 nAChRs during acute perfusion of increasing concentrations of strychnine.

We next examined [V13′T]α10 nAChR response to acute perfusion of a range of strychnine concentrations. The modulatory effect of strychnine had an inverted U-shaped concentration dependence (Fig. 9). At low concentrations (10 nM – 10 μM), strychnine enhanced [V13′T]α10 responses to ACh, while at the highest concentration (100 μM) strychnine produced an inhibitory effect. Maximum potentiation occurred at 1 μM strychnine, enhancing ACh-responses by greater than 5-fold at this concentration. ACh-evoked current responses were almost entirely inhibited by 100 μM strychnine.

## Discussion

The rules for assembly of multi-subunit transmembrane proteins remain under intense investigation. Based on the lack of nAChR function when mammalian α10 nAChR subunits are expressed alone, it has previously been assumed that α10 subunits do not co-assemble into functional homomeric receptors. The present study demonstrates that human α10 nAChR subunits will assemble as a homomer. We demonstrated this in two ways. First, wild-type subunits are expressed when co-injected with a well-known nAChR molecular chaperone, RIC-3. In the absence of RIC-3, the expression is small or absent, explaining why, for decades, mammalian homomeric α10 nAChRs have been thought not to express. However, the related mammalian α9 nAChR subunit will form a homomer, and chick α10 subunits will also form a homomer (5, 21), consistent with our finding that human α10 subunits can assemble into a homomer. The ACh-induced currents observed with wild-type α10 + RIC-3 are relatively small in amplitude (17 nA average, see Fig. 1) but were significantly greater than those observed in α10-only injected oocytes. No ACh-induced currents were observed in uninjected oocytes or oocytes injected with RIC-3 alone (Fig. 3*A*). Similarly, small currents have been reported for both human and rat homomeric α9 nAChRs (5, 22).

RIC-3 was originally identified in the soil nematode *Caenorhabditis elegans* and was subsequently shown to be conserved across eukaryotic organisms in the biological kingdom Animalia, where it facilitates the maturation and transport to the cell surface of Cys-loop receptors. Other non-α10-containing mammalian nAChR subtypes are difficult to express *in vitro*. For example, the α7 nAChR subunit expresses poorly or not at all in vertebrate expression cells but will express if combined with RIC-3. α7 and α10 nAChR subunits are present in immune cells, as is RIC-3 (18), suggesting that RIC-3 may facilitate α7 and/or α10 nAChR homomer assembly in these cells. Interestingly, however, mouse brain neuronal α7 nAChRs are functionally expressed in RIC-3 knock-out mice, indicating that RIC-3 is not essential for α7 expression in the CNS. To elucidate other potential α7 nAChR chaperones, a large-scale high-throughput screening approach was carried out by Gu *et al.*, who identified a four-pass transmembrane domain (TM) protein, NACHO, that enables homomeric α7 assembly in cell lines. Remarkably, knockout of the NACHO gene in mice eliminated functional α7 expression in CNS neurons, indicating the essential role of NACHO in this tissue (10). However, whereas NACHO expression is found in neurons, it is absent in immune cells such as macrophages (23). Thus, it is possible that in addition to RIC-3, there is a yet-to-be-elucidated molecular chaperone that would further enable α10 nAChR expression in immune cells. Such chaperones have been proposed to be important for α9α10 expression in cochlear hair cells and other receptor subtypes (24, 25).

The second way we demonstrated that α10 subunits will self-assemble was by using a reporter mutant. The transmembrane domain 2 (TM2) lines the channel pore of Cys-loop receptor neurotransmitter-gated ion channels. Residues within TM2 are involved in channel gating. Mutagenesis of hydrophobic residues that face the lumen of the pore has resulted in gain-of-function receptors. We exploited this feature to determine whether α10 subunits would form functional receptors independent of exposure to small molecule alkaloids. Mutation of a Val residue to Thr in the 13′ position was utilized as this mutation had previously been shown to have the largest effect when tested in the context of α9α10 heteromeric nAChRs (15). Injection of [V13′T]α10 resulted in functional expression, even in the absence of RIC-3. Co-injection of [V13′T]α10 and RIC-3 resulted in larger functional responses, which were further enhanced by exposure to strychnine. Mutations that facilitate the gating of the receptor may enhance the efficacy of a partial agonist or convert an antagonist into an agonist. It was previously demonstrated that nicotine is an antagonist of wild-type α9α10 nAChRs (5, 26). However, nicotine acts as a partial agonist of [V13′T]α10 homomers (Fig. 5).

The present study also demonstrates that the α10/α10 subunit interface is sufficient for agonists to evoke channel opening. This complements previous research that shows that the α10 subunit can serve as an additional ACh-binding site in α9α10 nAChRs that minimally have α9/α9, α10/α9 and α9/α10 subunit interface agonist binding sites (27). Interestingly, studies with a selective antagonist indicate that human α9α10 nAChRs can be present in different stoichiometries such as (α9)_2_(α10)_3_ and (α9)_3_(α10)_2_ (28). Given that ACh can activate α10 homomers by binding to the α10/α10 interface, ACh binding to an α10/α10 interface in α9α10 nAChRs may also occur. This also raises the possibility of other functional α9α10 nAChR stoichiometries, such as (α9)_1_(α10)_4._

The present study also indicates that exposure to certain small molecule ligands, including atropine and strychnine, rapidly increases the functionality of α10 homomeric receptors. nAChR assembly is tightly controlled and slow. The half-time of assembly of the muscle nAChR is more than 90 minutes (29). Thus, the rapid effects of atropine and strychnine are more likely to affect the functional state of the receptor. These alkaloid ligands might increase function by acting at the orthosteric ACh binding site to shift the receptor population equilibrium to one with an increased number of agonist-activatable receptors. Another possibility is that strychnine and/or atropine are acting at an allosteric site as a positive modulator. The effects of RIC-3 and strychnine are additive. Oocytes expressing α10 plus RIC-3 show a further substantial increase in expression if exposed acutely to lower concentrations of strychnine. Higher concentrations of strychnine block α10 nAChR function, suggesting the possibility that strychnine may have more than one binding site on the α10 nAChR, with one site that potentiates function (the higher affinity site) and one site that inhibits nAChR function (the lower affinity site). Strychnine is best known as an antagonist of pentameric glycine receptors but also blocks homomeric α7 nAChRs and homomeric α9 and α9α10 nAChRs (5, 30, 31). Notably, low-concentration strychnine acts to increase the ACh response of α10 homomers.

nAChRs in immune cells are part of the cholinergic anti-inflammatory pathway that serves to modulate the immune response to tissue injury and pathogens. Stimulation of the efferent vagus nerve releases ACh which interacts with nAChRs to regulate the release of inflammatory cytokines from macrophages and other cytokine-secreting immune cells (32); this interaction creates a communication interface between the brain and the immune system. Studies with knockout mice and selective antagonists have demonstrated that the nAChRs that mediate this response contain α7, α9, and/or α10 subunits (2). However, the specific subtypes and stoichiometries of these nAChRs are not precisely known. The present study suggests that α10 homomers could be among the receptors expressed by native cell populations. Although α10 homomers are sensitive to α-bungarotoxin, this snake toxin also blocks α7 homomers, α9α10 heteromers, and muscle type nAChRs. Thus, although α-bungarotoxin sensitivity is a characteristic of α10 homomers, this sensitivity is not unique to this nAChR subtype. Certain conopeptide analogs will selectively block α7 homomers and α9α10 heteromers. However, there is no selective antagonist for α10 homomers. Future studies of immune cell nAChRs may substantially benefit from the development of such compounds.

In conclusion, the present work establishes that human α10 nAChR subunits will assemble into functional homomers *in vitro*. This finding raises the possibility that such receptors also form *in vivo*, especially in tissues where the α9 subunit is absent and in tissues where α9 and/or α7 are present. The study also suggests that receptor subtypes composed of combinations of α1-α10, β1-β4, and/or δ, ε, or γ subunits that do not form when expressed alone may assemble *in vitro* and *in vivo* in the presence of an appropriate molecular chaperone. Thus, the number of nAChR subtypes may be much greater than presently known, and the selective expression of molecular chaperones may fine-tune the tissue distribution of these nAChR subtypes.

## Experimental procedures

### Human [V13′T]**α**10 subunit construction

Primers containing the desired base change were designed with 10 to 15 bases flanking the mutation on either side. The mutant primers were incorporated using Pfu Turbo DNA polymerase (Agilent Technologies). The PCR product was digested with DpnI to remove template DNA and transformed into 5-alpha competent cells (NEB). DNA was isolated using QiaPrep Spin Miniprep kit (Qiagen, Germantown, MD). The mutant DNA was sequenced by the Sequencing Core Facility at the University of Utah to ensure the presence of the desired mutation.

### cRNA preparation

Plasmid cDNA was linearized with the appropriate restriction enzyme. The linearized cDNA was transcribed with the Invitrogen mMessage mMachine T7 RNA polymerase kit (Thermo Fisher Scientific). The cRNA was purified using Qiagen RNeasy Mini kit and the concentration was determined with an Epoch Microplate Spectrophotometer (Biotek Instruments).

### Oocyte electrophysiology

The protocol for obtaining oocytes from *X. laevis* frogs was approved by the University of Utah’s Institutional Animal Care and Use Committee. The frogs, sourced from *Xenopus*1, were housed and cared for in an AAALAC-accredited facility by university staff. The functional expression of human nAChRs containing α9 or α10 subunits in *X. laevis* oocytes was assessed by TEVC electrophysiology as previously described (7). Briefly, oocytes were injected with cRNA and incubated in frog saline at 16 °C for 2 to 4 days. For experiments examining the effect of RIC-3 on α10 and [V13′T]α10 expression, the oocytes were injected with cRNAs for human nAChR subunits and RIC-3 at a 2:1 ratio, respectively (∼60 ng and 30 ng per oocyte). Oocytes expressing [V13′T]α10 nAChR were pulsed with ACh (100 μM) for 1 s every 90 s. Oocytes expressing α9 or α9α10 nAChRs were pulsed with ACh (100 μM) for 1 s at 60 s intervals. Oocytes were pulsed until a stable baseline response to ACh was achieved. Then, the saline solution was switched to one containing the ligand of interest for 5 min. For experiments examining the effects of atropine or strychnine on current amplitudes, the oocytes were perfused for 10 min with saline containing the alkaloid ligand under investigation. The final amplitude measured after exposure was normalized to the mean amplitude of the 3 ACh pulses prior to exposure. For experiments that utilized ligand concentrations ≥ 3 μM, the oocytes were incubated in a static bath for 5 min. When assessing the activity of agonists, oocytes were pulsed with increasing concentrations of ACh, choline, or nicotine (1 μM – 10 mM), and the current amplitudes were normalized to those produced by a 10 mM ACh pulse.

### Data and statistical analysis

All statistical analysis was conducted using Prism 10 (RRID: SCR_002798) (GraphPad Software). To assess differences in ACh-induced current amplitudes in oocytes expressing [V13′T]α10 *versus* [V13′T]α10 together with RIC-3, the data were first analyzed for normality using a Shapiro-Wilks test. Data sets determined to be normally distributed were assessed by a Student *t*-test for significance, and those not normally distributed were assessed using a Mann–Whitney test. Current amplitudes were normalized by the following procedure to account for expression variance among oocytes. For each oocyte, the amplitudes of the last 3 traces before ligand exposure were normalized to their combined mean (control group). The responses after ligand exposure were also normalized to this mean (experimental group). Individual points shown in the bar graphs are biological replicates. Concentration-response data for ACh, choline, and nicotine were analyzed utilizing the four-parameter nonlinear regression equation: Y = Bottom + (Top-Bottom)/ (1 + 10∧((LogEC50-X)∗HillSlope)).

### Materials

Human α9 and α10 subunits in the pSGEM vector containing the alfalfa mosaic virus sequence were obtained and prepared as previously described (22, 33). Human RIC3 cDNA in pcDNA3 was generously provided by William Green. RgIA-5474 and RgIA-4 were synthesized as previously described (13, 14). Acetylcholine chloride and dihydro-β-erythroidine hydrobromide were purchased from Tocris. Nicotine hydrogen tartrate salt, choline chloride, atropine sulfate salt monohydrate, α-bungarotoxin, and actinomycin D were purchased from Sigma Aldrich.

## Data availability

Raw data is available upon request to the primary author by email. Please send requests to bjtekarli@gmail.com.

## Conflict of interest

The authors declare that they have no conflicts of interest with the contents of this article.

**Table 1:** Comparison of agonist-induced currents in [V13′T]**α**10 nAChR expressing oocytes

Agonist conc.	Nicotine		Choline		Acetylcholine		Significance	
	Mean	SD	Mean	SD	Mean	SD	ACh, Ch	ACh, Nic
10 μM	1.8	1.6	7.8	8.1	16.7	9.5		∗**∗**
100 μM	2.9	0.2	13.0	5.5	25.3	8.4	∗	∗∗∗∗
300 μM	8.6	4.8	20.3	7.7	54.4	10.0	∗∗∗∗	∗∗∗∗
1 mM	13.7	5.4	31.9	8.8	74.5	11.7	∗∗∗∗	∗∗∗∗
3 mM	18.9	2.2	41.9	8.4	89.5	5.3	∗∗∗∗	∗∗∗∗

Statistical analysis of data shown in Fig. 5. Acetylcholine (ACh) was compared to choline (Ch), and acetylcholine was compared to nicotine (Nic). Values represent the mean percent response ± standard deviation. Percent response was calculated by considering the response to 10 mM ACh to be 100%. ∗*p* < 0.05, ∗∗*p* < 0.01, and ∗∗∗∗*p* < 0.0001 as calculated using an unpaired two-tailed *t*-test*.*

## References

[1] Zoli M., Pucci S., Vilella A., Gotti C. (2018). Neuronal and extraneuronal nicotinic acetylcholine receptors. Curr. Neuropharmacol..

[2] Richter K., Grau V. (2023). Signaling of nicotinic acetylcholine receptors in mononuclear phagocytes. Pharmacol. Res..

[3] Dani J.A. (2015). Neuronal nicotinic acetylcholine receptor structure and function and response to nicotine. Int. Rev. Neurobiol..

[4] Nelson M.E., Kuryatov A., Choi C.H., Zhou Y., Lindstrom J. (2003). Alternate stoichiometries of alpha4beta2 nicotinic acetylcholine receptors. Mol. Pharmacol..

[5] Elgoyhen A.B., Johnson D.S., Boulter J., Vetter D.E., Heinemann S. (1994). Alpha 9: an acetylcholine receptor with novel pharmacological properties expressed in rat cochlear hair cells. Cell.

[6] Marcovich I., Moglie M.J., Carpaneto Freixas A.E., Trigila A.P., Franchini L.F., Plazas P.V. (2020). Distinct evolutionary trajectories of neuronal and hair cell nicotinic acetylcholine receptors. Mol. Biol. Evol..

[7] Hone A.J., McIntosh J.M. (2022). Alkaloid ligands enable function of homomeric human alpha10 nicotinic acetylcholine receptors. Front Pharmacol..

[8] Kremiller K.M., Kulkarni G.C., Harris L.M., Gunasekara H., Kashyap Y., Ilktach G. (2024). Discovery of antinociceptive alpha9alpha10 nicotinic acetylcholine receptor antagonists by stable receptor expression. ACS Chem. Biol..

[9] Halevi S., McKay J., Palfreyman M., Yassin L., Eshel M., Jorgensen E. (2002). The C. elegans ric-3 gene is required for maturation of nicotinic acetylcholine receptors. EMBO J.

[10] Gu S., Matta J.A., Lord B., Harrington A.W., Sutton S.W., Davini W.B. (2016). Brain α7 nicotinic acetylcholine receptor assembly requires NACHO. Neuron.

[11] Peng H., Ferris R.L., Matthews T., Hiel H., Lopez-Albaitero A., Lustig L.R. (2004). Characterization of the human nicotinic acetylcholine receptor subunit alpha (alpha) 9 (CHRNA9) and alpha (alpha) 10 (CHRNA10) in lymphocytes. Life Sci..

[12] Hone A.J., McIntosh J.M. (2023). Nicotinic acetylcholine receptors: therapeutic targets for novel ligands to treat pain and inflammation. Pharmacol. Res..

[13] Romero H.K., Christensen S.B., Di Cesare Mannelli L., Gajewiak J., Ramachandra R., Elmslie K.S. (2017). Inhibition of α9α10 nicotinic acetylcholine receptors prevents chemotherapy-induced neuropathic pain. Proc. Natl. Acad. Sci. U. S. A..

[14] Gajewiak J., Christensen S.B., Dowell C., Hararah F., Fisher F., Huynh P.N. (2021). Selective penicillamine substitution enables development of a potent analgesic peptide that acts through a non-opioid-based mechanism. J. Med. Chem..

[15] Plazas P.V., De Rosa M.J., Gomez-Casati M.E., Verbitsky M., Weisstaub N., Katz E. (2005). Key roles of hydrophobic rings of TM2 in gating of the alpha9alpha10 nicotinic cholinergic receptor. Br. J. Pharmacol..

[16] Sobczak K., Bangel-Ruland N., Leier G., Weber W.-M. (2010). Endogenous transport systems in the Xenopus laevis oocyte plasma membrane. Methods.

[17] Miledi R., Parker I. (1984). Chloride current induced by injection of calcium into Xenopus oocytes. J. Physiol..

[18] Innocent N., Livingstone P.D., Hone A., Kimura A., Young T., Whiteaker P. (2008). Alpha-conotoxin Arenatus IB[V11L,V16D] [corrected] is a potent and selective antagonist at rat and human native alpha7 nicotinic acetylcholine receptors. J. Pharmacol. Exp. Ther..

[19] Whiteaker P., Christensen S., Yoshikami D., Dowell C., Watkins M., Gulyas J. (2007). Discovery, synthesis, and structure activity of a highly selective alpha7 nicotinic acetylcholine receptor antagonist. Biochemistry.

[20] Verbitsky M., Rothlin C.V., Katz E., Elgoyhen A.B. (2000). Mixed nicotinic-muscarinic properties of the alpha9 nicotinic cholinergic receptor. Neuropharmacology.

[21] Lipovsek M., Fierro A., Pérez E.G., Boffi J.C., Millar N.S., Fuchs P.A. (2014). Tracking the molecular evolution of calcium permeability in a nicotinic acetylcholine receptor. Mol. Biol. Evol..

[22] Filchakova O., McIntosh J.M. (2013). Functional expression of human α9∗ nicotinic acetylcholine receptors in X. laevis oocytes is dependent on the α9 subunit 5' UTR. PLoS One.

[23] Deshpande A., Vinayakamoorthy R.M., Garg B.K., Thummapudi J.P., Oza G., Adhikari K. (2020). Why does knocking out NACHO, but not RIC3, completely block expression of α7 nicotinic receptors in mouse brain?. Biomolecules.

[24] Gu S., Knowland D., Matta J.A., O’Carroll M.L., Davini W.B., Dhara M. (2020). Hair cell α9α10 nicotinic acetylcholine receptor functional expression regulated by ligand binding and deafness gene products. Proc. Natl. Acad. Sci..

[25] Gotti C., Clementi F., Zoli M. (2024). Auxiliary protein and chaperone regulation of neuronal nicotinic receptor subtype expression and function. Pharmacol. Res..

[26] Sgard F., Charpantier E., Bertrand S., Walker N., Caput D., Graham D. (2002). A novel human nicotinic receptor subunit, alpha10, that confers functionality to the alpha9-subunit. Mol. Pharmacol..

[27] Boffi J.C., Marcovich I., Gill-Thind J.K., Corradi J., Collins T., Lipovsek M.M. (2017). Differential contribution of subunit interfaces to α9α10 nicotinic acetylcholine receptor function. Mol. Pharmacol..

[28] Indurthi D.C., Pera E., Kim H.L., Chu C., McLeod M.D., McIntosh J.M. (2014). Presence of multiple binding sites on α9α10 nAChR receptors alludes to stoichiometric-dependent action of the α-conotoxin, Vc1.1. Biochem. Pharmacol..

[29] Green W.N., Claudio T. (1993). Acetylcholine receptor assembly: subunit folding and oligomerization occur sequentially. Cell.

[30] Matsubayashi H., Alkondon M., Pereira E.F.R., Swanson K.L., Albuquerque E.X. (1998). Strychnine: a potent competitive antagonist of α-bungarotoxin-sensitive nicotinic acetylcholine receptors in rat hippocampal neurons. J. Pharmacol. Exp. Ther..

[31] Elgoyhen A.B., Vetter D.E., Katz E., Rothlin C.V., Heinemann S.F., Boulter J. (2001). alpha10: a determinant of nicotinic cholinergic receptor function in mammalian vestibular and cochlear mechanosensory hair cells. Proc. Natl. Acad. Sci. U. S. A..

[32] Pavlov V.A., Wang H., Czura C.J., Friedman S.G., Tracey K.J. (2003). The cholinergic anti-inflammatory pathway: a missing link in neuroimmunomodulation. Mol. Med..

[33] Azam L., McIntosh J.M. (2012). Molecular basis for the differential sensitivity of rat and human α9α10 nAChRs to α-conotoxin RgIA. J. Neurochem..

